# The Reply of “Trinity University”

**Published:** 1904-05

**Authors:** 


### Editorial.

### THE REPLY OF "TRINITY UNIVERSITY."

The International of April contained a "Reply from 'Trinity University,' Toronto," Canada, in answer to a charge made in the February number that this University was graduating men, with the degree of Doctor of Dental Surgery, upon examination. This reply from the pen of the Secretary of the Board of Dental Studies, Trinity University, does not seem to the writer to meet satisfactorily the issue as presented.

The main question is, Does the Trinity University grant the dental degree upon an examination? While this is not denied, it is met in a way that leaves the reader to infer that lectures are the all-important method of training in dentistry. The Secretary says, "A careful study of the curriculum . . . will, I feel sure, lead you to recognize that the knowledge required to obtain a D.D.S. from this University is equal to that required by the most advanced dental institutions on this continent." This very broad statement needs confirmation, and the published curriculum should show it if it exists.

The following quotations are made from the "Extract from the University Calendar" giving "the regulations governing the degree of D.D.S." After stating terms of admission, the candidate "must pass three University examinations, called the First, Second, and Third Examinations in Dentistry."

"Before admission to the First Examination he must have attended, in some dental college, recognized by this University, one course of lectures on the following subjects: Chemistry, Anatomy, Histology, Bacteriology, Comparative Dental Anatomy, Technique, Metallurgy." The subjects of the first examination are then given in detail.

For the Second Examination the candidate must have attended "lectures on the following subjects: Operative Dentistry, Prosthetic Dentistry, Anatomy, Physiology, Chemistry, Medicine and Surgery, Materia Medica." It will be interesting to practical men to know the extent of the examination in Operative Dentistry. It comprises "Development of the teeth, clinical history and pathology of caries, pulpitis, pericementitis, and alveolar abscess, the composition and preparation of materials for filling teeth." Subsequently it is stated that "Candidates will be required to satisfy the examiners in Practical Dentistry as to Operative and Prosthetic work, crown- and bridge-work and Orthodontia, either by practical work done under their supervision or by certificate from a recognized dental college."

The Third, or Final, Examination covers about all the subjects enumerated in the First and Second Examinations. The following concludes this matter: "Candidates who present certificates of having passed the First and Second Year Examinations, in some recognized dental college or university, will be admitted to third year standing." Farther on we find the following remarkable paragraph, heretofore quoted in original article. "*All legally qualified* practitioners of dental surgery of Great Britain, or of any portion of the British Empire, *will be admitted to the Final Examination without passing further matriculation and without further attendance on lectures.*" (Italics ours.)

It must be evident from these quotations that the assertion that the "knowledge required to obtain a D.D.S. from this University [Trinity] is equal to that required by the most advanced dental institutions on this continent" is only true in theory. Nothing practical is taught, and certificates as to proficiency in practical matters are accepted. The writer is forced to the conviction that this school is not only *not* equal to others on this continent, but is *not a dental school in any sense and is not deserving of that title.*

The Secretary tries very hard to modify the language of the paragraph quoted, and seems to think it "does not make the degree easy to obtain, nor does it call for a low standard of knowledge on the part of the candidates." He acknowledges that "The qualification of which the candidates must furnish proof in order to be admitted to the Final Examination, is that he has been licensed by the government of his own country for the practice of dental surgery, and that that country be a part of the British Empire." Then he proceeds to state, "As a matter of fact all parts of the empire, with only one or two minor exceptions, require regular apprenticeship and a College Course in order that a candidate may obtain his license to practise." Exactly where the "minor exceptions" are to be found he is careful not to mention. Attention was called in our original statement to one portion of the British Empire, and by no means an inconsiderable portion,—that of New Zealand,—where no dental college exists and where a state examination is alone required. If we are not mistaken, there are already at Trinity, or in the neighborhood preparing for the final examination, a candidate or candidates from that country, who will, doubtless, be given the D.D.S. under the ruling stated. The writer is not familiar with the status of affairs in South Africa relating to dental training, but the inference may be drawn that the acquisition of this portion of the empire has been too recent to establish any positive methods in dental training beyond the so-called apprenticeship system.

The secretary lays special stress upon the fact that the candidate, upon receiving the degree of D.D.S., must promise not to resort to any unethical methods, and if at any time it can be shown that he has violated this promise "in any way," he agrees, upon demand, to surrender his diploma and have his name stricken from the rolls.

This seems to require an unbounded faith in human nature, but from experience the writer is of the opinion that the promise will be found to be hardly worth the time expended in exacting it from the candidate.

The writer holds that this method of conferring degrees is wrong in principle and worse in practice. It is a reiteration of the old idea, that no matter where a person has secured his knowledge, he is entitled to present the evidence in order to secure a degree. This, in theory, seems absolutely just and right; but in practice it had been found to be the chief promotor of all the fraudulent diploma traffic of this and other countries. The word examination may mean much or little, depending on who makes it and how it is made.

The secretary wonders how the writer came to the idea that the recipient (of this degree) is not to make use of it in the Dominion of Canada. It is due to "Trinity" to say that it is not responsible for this portion of the original charge. The secretary will find, on page 10 of the Announcements of the Royal College of Dental Surgery, a paragraph containing the following words. This was quoted in the February number, but must have been overlooked. It is repeated here: "Students of dentistry *not desiring to qualify for practice in the Province of Ontario,* by obtaining the L.D.S., of the R.C.D.S. of Ontario, may avail themselves of all the privileges of the School of Dentistry of the R.C.D.S., and proceed to the degree of Doctor of Dental Surgery, either in the University of Toronto, or in Trinity University, or compliance with the requirements of their Curriculum in Dentistry." (Italics ours.) In plain English this means, to the writer's comprehension, that the degree of D.D.S., as granted by "Trinity," does not qualify for practice in Ontario. If this be not the correct interpretation, then it is time the scribe who prepared the Announcement of the Royal College of Dental Surgeons should change the construction of the sentence, for the writer is not alone in his interpretation.

The secretary acknowledges that after the first day of October next the Trinity University will be united with the University of Toronto and "exemptions allowed to regularly qualified practitioners of dental surgery within the British Empire will then cease to be operative," and we tender our congratulations that a definite period has been fixed when this very objectionable method will come to an end with our good neighbors over the border.

